# *Fagopyrum esculentum* ssp. *ancestrale-*A Hybrid Species Between Diploid *F. cymosum* and *F. esculentum*

**DOI:** 10.3389/fpls.2020.01073

**Published:** 2020-07-16

**Authors:** Cheng Cheng, Yu Fan, Yu Tang, Kaixuan Zhang, Dinesh C. Joshi, Rintu Jha, Dagmar Janovská, Vladimir Meglič, Mingli Yan, Meiliang Zhou

**Affiliations:** ^1^ Institute of Crop Sciences, Chinese Academy of Agricultural Sciences, Beijing, China; ^2^ School of Life Sciences, Hunan University of Science and Technology, Xiangtan, China; ^3^ Department of Tourism, Sichuan Tourism University, Chengdu, China; ^4^ Indian Council of Agricultural Research- Vivekananda Institute of Hill Agriculture, Almora, India; ^5^ Gene Bank, Crop Research Institute, Prague, Czechia; ^6^ Crop Science Department, Agricultural Institute of Slovenia, Ljubljana, Slovenia

**Keywords:** buckwheat, chloroplast genome, *Fagopyrum esculentum* ssp. *ancestrale*, molecular markers, phylogenetics

## Abstract

*Fagopyrum cymosum* is considered as most probable wild ancestor of cultivated buckwheat. However, the evolutionary route from *F. cymosum* to *F. esculentum* remains to be deciphered. We hypothesized that a hybrid species exists in natural habitats between diploid *F. cymosum* and *F. esculentum*. The aim of this research was to determine the phylogenetic position of *F. esculentum* ssp. *ancestrale* and to provide new thoughts on buckwheat evolution. Different methodologies including evaluation of morphological traits, determination of secondary metabolites, fluorescence *in situ* hybridization (FISH), comparative chloroplast genomics, and molecular markers were deployed to determine the phylogenetic relationship of *F. esculentum* ssp. *ancestrale* with *F. cymosum* and *F. esculentum*. The ambiguity observed in morphological pattern of genetic variation in three species revealed that *F.* *esculentum* ssp. ancestrale is closely related to *F. cymosum* and *F. esculentum*. Flavonoid analysis revealed that *F.* *esculentum* ssp. *ancestrale* is closely related to *F. esculentum*. Comparative chloroplast genome analysis further supported the close proximity of *F. esculentum* ssp. *ancestrale* with *F. esculentum*. Additionally, molecular marker analysis revealed that *F. esculentum* ssp. *ancestrale* exhibits co-dominance with the bands amplified by *F. cymosum* and *F. esculentum*. These finding provided supporting evidence in favor of the hypothesis that *F. esculentum* ssp. *ancestrale* is a hybrid species between *F. cymosum* to *F. esculentum*, which was probably originated by spontaneous hybridization under natural conditions.

## Introduction

Buckwheat (*Fagopyrum* spp.) originated in southwestern China is one of the oldest domesticated crops from Asia. Out of the 26 known species of genus *Fagopyrum*, that includes diploids (2n =2x= 16) and tetraploids (2n = 4x = 32), *F. esculentum* (common buckwheat) and *F. tataricum* (tartary buckwheat) are the two cultivated species (Joshi et al., 2020). Due to the alterable (ordered) position of *Fagopyrum* in family *Polygonaceae*, it was difficult to deduce the evolutionary patterns and phylogenetic relationships of *Fagopyrum* species for a long time (Ohnishi, 2016). In order to understand the complex phylogenetic relationships and domestication events, *Fagopyrum* species are classified into two groups based on kernel morphology: the big achene group (*cymosum* group) and the small achene group (*urophyllum* group) (Ohnishi and Matsuoka, 1996). Except for kernel morphology, two diploid varieties *F. cymo*sum var. *pilus* and *F. cymosum* var. *megaspartanium* of the large achene group were very similar to the two cultivated species in many aspects and were considered the potential ancestors of common buckwheat (*F. esculentum*) and tartary buckwheat (*F. tataricum*) respectively (Steward, 1930; Ohnishi, 2016). However, reports based on morphological cladistics, isozyme analysis, and DNA polymorphism suggested that *F. cymosum* is not the direct ancestor of buckwheat; it is only distantly related to *F. tataricum* and *F. esculentum* (Ohnishi and Matsuoka, 1996; Kadyrova et al., 2010; Zhou et al., 2012).

To further resolve the issue of buckwheat evolution, Ohnishi (1991; 1998) proposed two wild species; *F. esculentum* ssp. *ancestrale* from Yunan province and *F. tataricum* ssp. *potanini* from Sichuan province of China as probable ancestors of *F. esculentum* and *F. tataricum* respectively. The two candidate species were considered as wild progenitors of cultivated species because of their differences in dormancy and shattering habit that are mainly used to make a distinction between cultivated species and its close wild relatives (Ohnishi and Matsuoka, 1996).

During our germplasm expedition undertaken from the year 2016–2018, we found a dense distribution of *F. esculentum* ssp. *ancestrale* populations in Yunnan province of China. Upon examining the morphological pattern of genetic variation, it was observed that wild populations of *F. esculentum* ssp. *ancestrale* are quite similar to *F. cymosum* and *F. esculentum* raising the hypothesis that *F. esculentum* ssp. *ancestrale* is a hybrid species between *F. cymosum* and *F. esculentum*. However, due to their strong dependency on environment and stage specific expression, morphological parameters are not the most reliable and informational entities in taxonomic and phylogenetic studies. Therefore, in the present paper, we describe the findings of subsequent experiments based on secondary metabolites (flavonoid) analysis, fluorescence *in situ* hybridization (FISH), comparative chloroplast genomics and DNA polymorphism performed to define the phylogenetic relationships of *F. esculentum* ssp. *ancestrale* with *F. cymosum* and *F. esculentum.* The FISH with total nuclear and ribosomal DNA as probes is an easy and robust method of molecular cytogenetics to physically map and compare buckwheat genomes (Walling et al., 2013), which may provide visible karyotypes of three buckwheat species under study. Moreover, chloroplast genomes are considered as a potentially useful tool in plant evolutionary studies, because of the non-meiotic and mostly uniparental inheritance of genes. The comparative analysis of chloroplast genomes indicated that the *Fagopyrum* possesses some unique structural features including the InDel markers that can define the evolutionary relationships of different species (Logacheva et al., 2008; Cho et al., 2015). Finally, robust microsatellite markers rendered them as the most reliable and informational entities to complement morphological descriptors in buckwheat taxonomic and phylogenetic studies. This study contributes to new insights of buckwheat evolution.

## Materials and Methods

### Plant Materials

A total of 22 accessions belonging to 15 *Fagopyrum* species comprising thirteen wild buckwheat species including *F. esculentum* ssp. *ancestrale* and two cultivated species (*F. esculentum* and *F. tataricum*) were used in the study (**Table S1**). These accessions were collected from different regions of Yunnan, Sichuan, and Beijing in China during the year 2016 to 2018. Out of the total 15 species enlisted in **Table S1**, *F. esculentum* ssp. *ancestrale*, *F. cymosum-Luojishan* and cultivated *F. esculentum-Yuqiao* were used for chromatography, FISH, chloroplast genome analysis, and molecular marker analysis, while the remaining twelve species were utilized for phylogenetic analysis using three molecular markers: ITS, *mat*K, and *trnH-psbA*.

### Chromatography Analysis

The buckwheat seeds dried to consistent weight were pulverized and sieved through a 40 mesh. The powdered sample (0.2 g) mixed with 10 ml 80% methanol in an Erlenmeyer flask was incubated in an ultrasonic cleaner at 40 khz and 45°C for 30 min and then passed the 0.45 μm filter. The test sample was the mixture of 1 ml filtrate, 2 ml aluminum trichloride (0.1 mol L^−1^), and 3 ml potassium acetic acid (1 mol L^−1^ and 4 ml methanol (80%, v/v). High performance liquid chromatography (HPLC) was used to measure the content of rutin and quercetin (Zhang et al., 2018). The total flavonoid content was determined by the aluminum trichloride method (colorimetric wavelength 420 nm) (Li et al., 2008).

### Fluorescence In Situ Hybridization (FISH) Analysis

The buckwheat seeds were submerged in water for 6 h at room temperature and then at 4 °C for 1 day, and afterwards sprouted at a consistent temperature of 25°C until the root tip was 1–2 cm long. The root tips were treated with nitrous oxide at 10 atm for 2 h in 1.5 ml Eppendorf tubes, incubated in 90% acetic acid for 8 min, and washed with ultrapure water for three times. The root tips were digested with cellulase and pectinase at 37°C for 90 min and washed with 70% ethanol for three times on ice. The dissecting needle was used to smash the root tip in 30 μl of 70% ethanol, centrifuged at low speed for 30–60 s. After discarding the supernatant, re-suspend with 30 μl acetic acid for 5 min. The suspension of 5–7 μl was used to determine the karyotypes by the microscopy observation (Stebbins, 1971; Hsiao et al. 1986). The 25S rDNA probe was generated by nick translation of a 2.3 kb *Cla*I subclone of the 25S rDNA coding region of *Arabidopsis thaliana* as described by Jenkins and Hasterok (2007). This probe was labeled with digoxigenin-11-dUTP (Roche) and used to visualize 45S rDNA loci containing the genes coding for 18S, 5.8S, and 25S rRNA. Pictures were captured by the OLYMPUS AX80 microscopy (Olympus Corporation, Japan) with a CCD camera (Diagnostic Instruments, USA).

### DNA Extraction, Sequencing, and Assembly

The total genomic DNA was extracted from 100 mg of fresh leaves by the modified CTAB method (Li et al., 2013). Subsequently, the total DNA was disrupted by ultrasound to produce fragments of 300–500 bp and the fragment quality was checked using Bioanalyzer 2100 (Agilent Technologies). A 400 bp DNA library was constructed using the NEB Next Ultra™ DNA Library Kit (Illumina, San Diego, California, USA). To assemble the chloroplast genome, we firstly using Hiseq 4000 PE 150 (Illumina, San Diego, California, USA) to sequencing the library fragments, then SPAdes (Bankevich et al., 2012) was used for *de novo* assemblies, the contigs obtained were further screened by BLAST, after that, Sequencher 4.10 (http://www.genecodes.com) were used to marge the screened contigs, finally, Geneious 8.1 (Kearse et al., 2012) was used to compare all reads to the spliced chloroplast genome sequence to test whether the counting sequence was correct or not.

### Chloroplast Genome Analysis

The chloroplast genome annotation of *F. cymosum*, *F. esculentum* and *F. esculentum* sp. *Ancestrale* was performed using DOGMA software (Wyman et al., 2004). BLASTX and BLASTN programme were utilized to search for the location of coding genes, transfer RNAs, and ribosomal RNAs. Due to its limitations, BLAST cannot annotate short exons as a result of which some exon intron regions were not well represented in cp genome. To overcome this, we made precise adjustments for the annotation based on other published chloroplast genome information. The circular genome maps were drawn by Organellar Genome DRAW (http://ogdraw.mpimpgolm.mpg) (Lohse et al., 2013) and edited by Adobe Illustrator CS5. The microsatellites (SSRs) were searched in cp genome using the Misa-web programme (Beier et al., 2017; https://webblast.ipk-gatersleben.de/misa/). The parameter (unit size/minimum number of repeats) utilized for the search were ten repeat units for mononucleotide microsatellites, six units for dinucleotide microsatellites and five repeat units each for tri, tetra, penta, and hexanucelotide microsatellites. The cp genomes of five buckwheat species (*F. cymosum*, *F. tataricum, F. esculentum*, *F. esculentum* sp. *ancestrale*, *F. lujojishanense*) were compared using m VISTA programme (Frazer et al., 2004) to reflect the unique characteristics of species. The five chloroplast genomes were compared using MAFFT v5 software (Katoh and Standley, 2013) with the default parameter settings for the alignment process, following with manual sequence adjustment by Seal (http://tree.bio.ed.ac.uk/software/seal.html). The principle of comparison was to open the inversions that appear in the sequence to avoid erroneous data polymorphism. The LSC, SSC and IR regions of the five species were calculated using the DnaSP v5.0 software. The sequence information of the five species were presented in **Table 1**.

**Table 1:** Comparison of the complete chloroplast genome contents of five *Fagopyrum* species.

GenBank accession number	*F. cymosum*	*F. luojishannse*	*F. esculentum*	*F. tataricum*	*F. esculentum* ssp. *ancestrale*
	KY275181	KY275182	MT572345	KM201427	MT572344
Total sequence length	159,320	159,265	159,576	159,272	159,600
Large signal copy (LSC)	84,422	84,431	84,875	84,398	84,892
Small signal copy (SSC)	13,264	13,094	13,331	13,292	13,334
Inverted repeat region (IR)	30,817	30,870	30,685	30,791	30,687
Total number of genes	114	114	113	114	113

### Phylogenetic Analysis

Phylogenetic analysis was performed on cp genome sequences of *Fagopyrum* species and other species of four closely related dicotyledonous genra (**Figure 7**). In this study, we used nine species belonging to *Polygonaceae* families, including five species of *Fagopyrum* (*F. cymosum*, *F. tataricum, F. esculentum*, *F. esculentum* sp. *ancestrale*, *F. lujojishanense*) for investigating the phylogenetic relationships of *Fagopyrum* species. The remaining four species belonging to genus *Rumex* or genus *Rheum* were used as outgroups. The chloroplast genome information and nucleotide sequence data were obtained from NCBI (**Table S2**). The phylogenetic tree based on a maximum likelihood method was constructed using RA x ML v7.2.8 (Stamatakis, 2006) with a bootstrap value of 1,000.

### Molecular Markers Analysis

Molecular marker analysis was performed on genomic DNA isolated from the leaves of *F. esculentum* ssp. *ancestrale*, *F. esculentum* and *F. cymosum*. The nucleotide sequences of SSR markers and PCR cycling conditions are furnished in **Supplementary Table S2**.

### Statistical Analysis

The results of total flavonoid content determination and chromosome arm ratio measurement presented in this article were based on the average of three parallel experiments. The data were analyzed with appropriate methods with the SPSS software (SPSS v. 25.0; IBM Co., Armonk, NY, USA). The statistical treatment was performed using Student’s t-test to examine the significant differences at a significance level of *P < 0.01.* Data mapping was processed using Origin 8.0 software (Origin Lab vol. 9.1).

## Results

### *F. esculentum* ssp. *ancestrale* Is Morphologically Related to *F. cymosum* and *F. esculentum*

**Figure 1:**
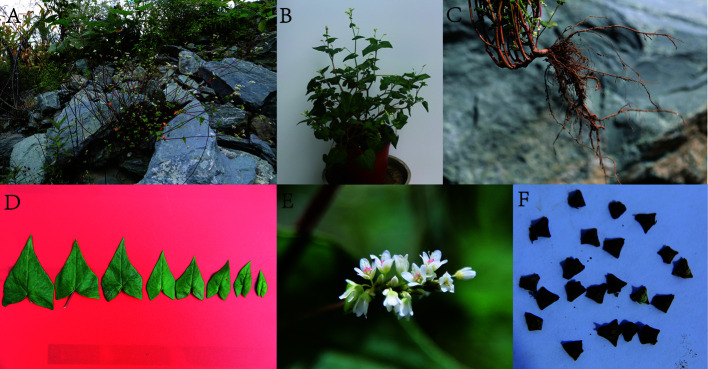
The morphological characters of *F. esculentum* ssp. *ancestrale*. A: Habitat, B: Individual pattern, C: Root, D: Leaf, E: Flower, F: Seed.

Phylogenetic relationships of *F. esculentum* ssp. *ancestrale* with *F. cymosum* and *F. esculentum* based on morphological characters are discussed in this section. A perusal of morphological characters revealed that differences among three buckwheat species occur for plant height, leaf morphology, perianth color and fruit shape (**Figure 1**, **Table 2**). For instance, perianth of *F. esculentum* ssp. *ancestrale* was characterized by white color whereas; *F. cymosum* and *F. esculentum* possess either white or pink colored perianth. Similarly, *F. esculentum* ssp. *ancestrale* can be distinguished by the presence of long triangular shaped achenes. In contrast, *F. cymosum* and *F. esculentum* were characterized by ovate shaped achenes. On account of leaf morphology, growth habit, inflorescence type, and seed color, all the three species appeared to be closely related to each other and no distinction can be made among them (**Table 2**). The ambiguity observed in morphological traits of *F. esculentum* ssp. *ancestrale*, *F. cymosum* and *F. esculentum* supported our hypothesis that *F. esculentum* ssp. *ancestrale* is a hybrid species between *F. cymosum* and *F. esculentum*.

**Table 2:** Morphological characters of *F. cymosum*, *F. esculentum* ssp. *ancestrale* and *F. esculentum*.

Index	*F. cymosum*	*F. esculentum* ssp. *ancestrale*	*F. esculentum*
Origin	Cultivar	Wild	Cultivar
Plant height (cm)	50~200 cm	50.5~154.0 cm	40~80 cm
Plant type	mostly erect, sometimes semi-erect	semi-erect	erect
Stem color	green or red-brown	green or red-brown	green or red
Leaf morphology	Triangular, Leaf width3-11 cm, leaf length 4-12cm	triangular, long oval and arrow shaped Leaf width 0.4~12.4 cm, leaf length 1.4~11 cm	triangular or ovate triangular, Leaf width 2-5cm, leaf length 2.5-7 cm
Petioles	leaves in upper part of stem have no petioles or short petioles	leaves in upper part of stem have no petioles or short petioles	leaves in upper part of stem have no petioles or short petioles
Inflorescence type	Capitate, axillary and terminal	Capitate, axillary and terminal	Capitate, axillary and terminal
Perianth number and color	5, white or pink	5, white	5, white or pink
Number of stamens	8	8	8
Number of style	3	3	3
Flower type diversity	Homomorphic flower	Homomorphic or Heteromorphic flower	Heteromorphic flower
Fruit color	Black-brown, no lustrous	Black-brown, no lustrous	Black-brown, no lustrous
Fruit type and shape	Achene, Broadly ovate	Achene, Long triangular	Achene, Ovate

### Flavonoid Pattern Confirmed Phylogenetic Relationship of *F. esculentum* ssp. *ancestrale* With *F. esculentum* and *F. cymosum*

**Figure 2:**
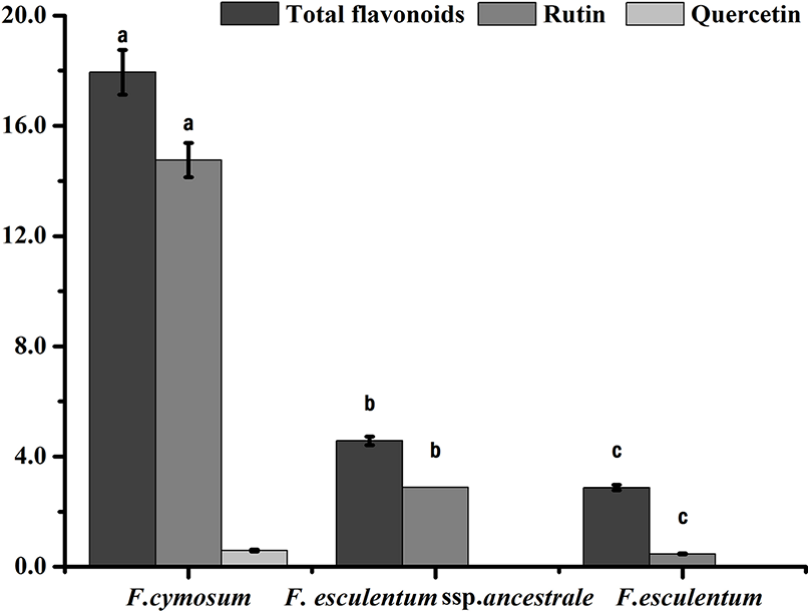
Flavonoids analysis of *F. esculentum* ssp. *ancestrale*, *F. cymosum* and *F. esculentum*. Error bars represent the standard deviation from three biological replicates determined by a Studentt’s t test (P < 0.01).

The total flavonoids, rutin and quercetin content of *F. esculentum* ssp. *ancestrale*, *F. cymosum* and *F. esculentum* were determined. *F. cymosum* had the highest total flavonoid content (17.9 mg g^-1^) followed by *F. esculentum* ssp. *ancestrale* (4.6 mg g^-1^) and *F. esculentum* (2.9 mg g^-1^). Likewise, significant differences were observed for the rutin content of *F. cymosum* (14.7 mg g^-1^) compared to the *F. esculentum* ssp. *ancestrale* (2.9 mg g^-1^) and *F. esculentum* (0.5 mg g^-1^) (**Figure 2**). While quercetin content was observed in trace quantity (0.6 mg g^-1^) in *F. cymosum*, it was absent in *F. esculentum* ssp. *ancestrale* and *F. esculentum*. Overall, it was observed that *F. esculentum* ssp. *ancestrale* is more closely related to *F.* *esculentum* compared to *F.* *cymosum* based on total flavonoids, rutin and quercetin contents.

### Cytogenetic Analysis Revealed Diploid Genomic Constitution of *F. esculentum* ssp. *ancestrale* and Its Close Proximity With *F. esculentum*

**Figure 3:**
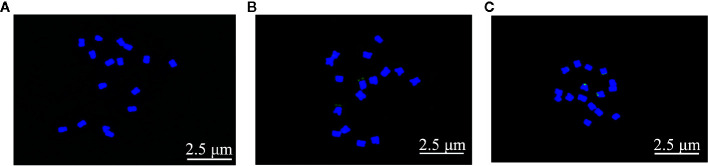
Cytogenetic analysis of *F. cymosum* **(A)**, *F. esculentum* ssp. *ancestrale* **(B)**, and *F. esculentum* **(C)**. The 25s fluorescent mark showed that there were 6 fluorescently named chromosome closes in both *F. esculentum* and *F. esculentum* ssp. *ancestrale*. At least three biological replicates of **(A**–**C)** were performed with the same results.

The mitotic chromosome analysis through FISH revealed diploid (2n=2x=16) genomic constitution of all the three species under investigation (**Figure 3**). The arm ratio of *F. esculentum* ssp. *ancestrale* and *F. esculentum* was from 1.033 to 1.563 and from 1.119 to 1.406 respectively, suggesting that their chromosome types were quite similar (2n=2x=16m) (Levan et al., 1964). Whereas, the *F. cymosum* was characterized as 2n=2x=15m+1sm by the presence of fifteen metacentric chromosomes (arm ratio 1.063–1.744) and one sub-metacentric chromosome (arm ratio 1.744). The ratio of the longest to the shortest chromosome of *F. cymosum*, *F. esculentum* ssp. *ancestrale* and *F. esculentum* was 1.967, 1.561, and 2.267.

### Chloroplast (cp) Genomics of *F. esculentum* and *F. esculentum* ssp. *ancestrale*

**Figure 4:**
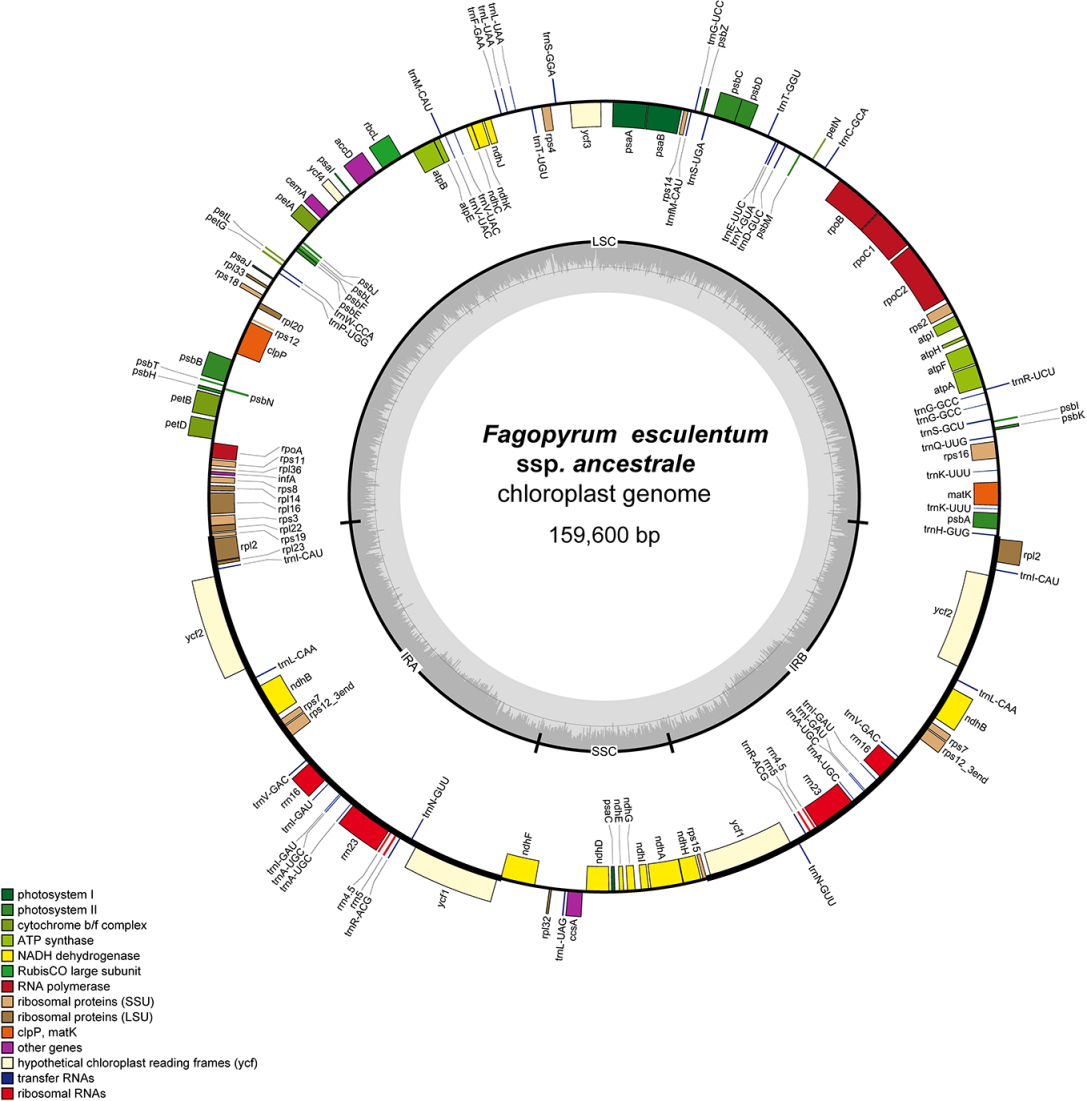
Gene map of *F. esculentum* ssp. *ancestrale* chloroplast genome. The genes shown outside of the circle are transcribed clockwise, while those inside are counter clockwise.

The cp genome of *F. esculentum* produced a total number of 0.58 million pair-end reads with 42.63 Gb of clean data. The size of the complete cp genome was 159,576 bp, which displayed a typical quadripartite structure, including a pair of inverted repeat region (2IRR; 30,685 bp) separated by the large single copy (LSC; 84,875 bp) and small single copy (SSC; 13,331 bp) regions (**Figure S1**, **Table 1**). Likewise, the cp genome of *F. esculentum* ssp. *ancestrale* was represented by the size of 159,600 bp and when compared with cp genome of *F. esculentum* it also displayed a typical quadripartite structure including a pair of inverted repeat region 2 IRR (30,687 bp) separated by the LSC (84,892 bp) and small single copy SSC (13,334 bp) regions (**Figure 4**, **Table 1**).

Gene annotation studies revealed that the cp genome of *F. esculentum* ssp. *ancestrale* contains 113 unique genes (**Table 3**), of which 79 are protein-coding (69.9%), 30 are transfer RNAs (26.6%), and 4 are ribosomal RNAs (3.5%). Further, the functional analysis divided the 113 genes into three categories including 60 transcription and translation regulating genes (53.0%), 47 genes related to photosynthesis (41.6%) and 6 genes with unknown function (5.4%). Half of the transcription and translation regulating genes are the transfer RNA genes and most of the Photosynthesis related genes belong to the photosystem II (31.9%) and NADPH dehydrogenase (23.4%). In total, we found 17 intron-containing genes, including fifteen genes contain one intron, and two genes (ycf3 and clpP) contain two introns. Rps12 is a specific trans-shear gene with its 5’ exon in the LSC region and the 3’ exon in the IR region (**Table 3**).

**Table 3:** Gene annotation and classification of chloroplast genome.

Gene category	Group of gene	Name of gene
Photosynthesis related genes	Rubisco	*rbcL*
	Photosystem I	*psaA*, *psaB*, *psaC*, *psaI*, *psaJ*
	Assembly/stability of photosystem I	*ycf3, *ycf4*
	Photosystem II	*psbA*, *psbB*, *psbC*, *psbD*, *psbE*, *psbF*, *psbH*, *psbI*, *psbJ*, *psbK*, *psbL*, *psbM*, *psbN*, *psbT*, *psbZ*
	ATP synthase	*atpA*, *atpB*, *atpE*, *atpF, *atpH*, *atpI*
	cytochrome b/f compelx	*petA*, *petB, *petD, *petG*, *petL*, *petN*
	cytochrome c synthesis	*ccsA*
	NADPH dehydrogenase	*ndhA, *ndhB, *ndhC*, *ndhD*, *ndhE*, *ndhF*, *ndhG*, *ndhH*, *ndhI*, *ndhJ*, *ndhK*
Transcription and translation related genes	transcription	*rpoA*, *rpoB*, *rpoC1*, *rpoC2*
	ribosomal proteins	*rps2*, *rps3*, *rps4*, *rps7*, *rps8*, *rps11*, *rps12, *rps14*, *rps15*, *rps16*, *rps18*, *rps19*, *rpl2, *rpl14*, *rpl16, *rpl20*, *rpl22*, *rpl23*, *rpl32*, *rpl33*, *rpl36*
	translation initiation factor	*infA*
RNA genes	ribosomal RNA	*rrn5*, *rrn4.5*, *rrn16*, *rrn23*
	transfer RNA	*trnA-UGC, *trnC-GCA*, *trnD-GUC*, *trnE-UUC*, *trnF-GAA*, *trnG-UCC*, *trnG-GCC, *trnH-GUG*, *trnI-CAU*, *trnI-GAU, *trnK-UUU, *trnL-CAA*, *trnL-UAA, *trnL-UAG*, *trnfM-CAUI*, *trnM-CAU*, *trnN-GUU*, *trnP-UGG*, *trnQ-UUG*, *trnR-ACG*, *trnR-UCU*, *trnS-GCU*, *trnS-GGA*, *trnS-UGA*, *trnT-GGU*, *trnT-UGU*, *trnV-GAC*, *trnV-UAC, *trnW-CCA*, *trnY-GUA*
Other genes	RNA processing	*matK*
	carbon metabolism	*cemA*
	fatty acid synthesis	*accD*
	proteolysis	*clpP
Function unknown	conserved reading frames	*ycf1*, *ycf2*

Intron-containing genes are marked by asterisks (*).

**Figure 5:**
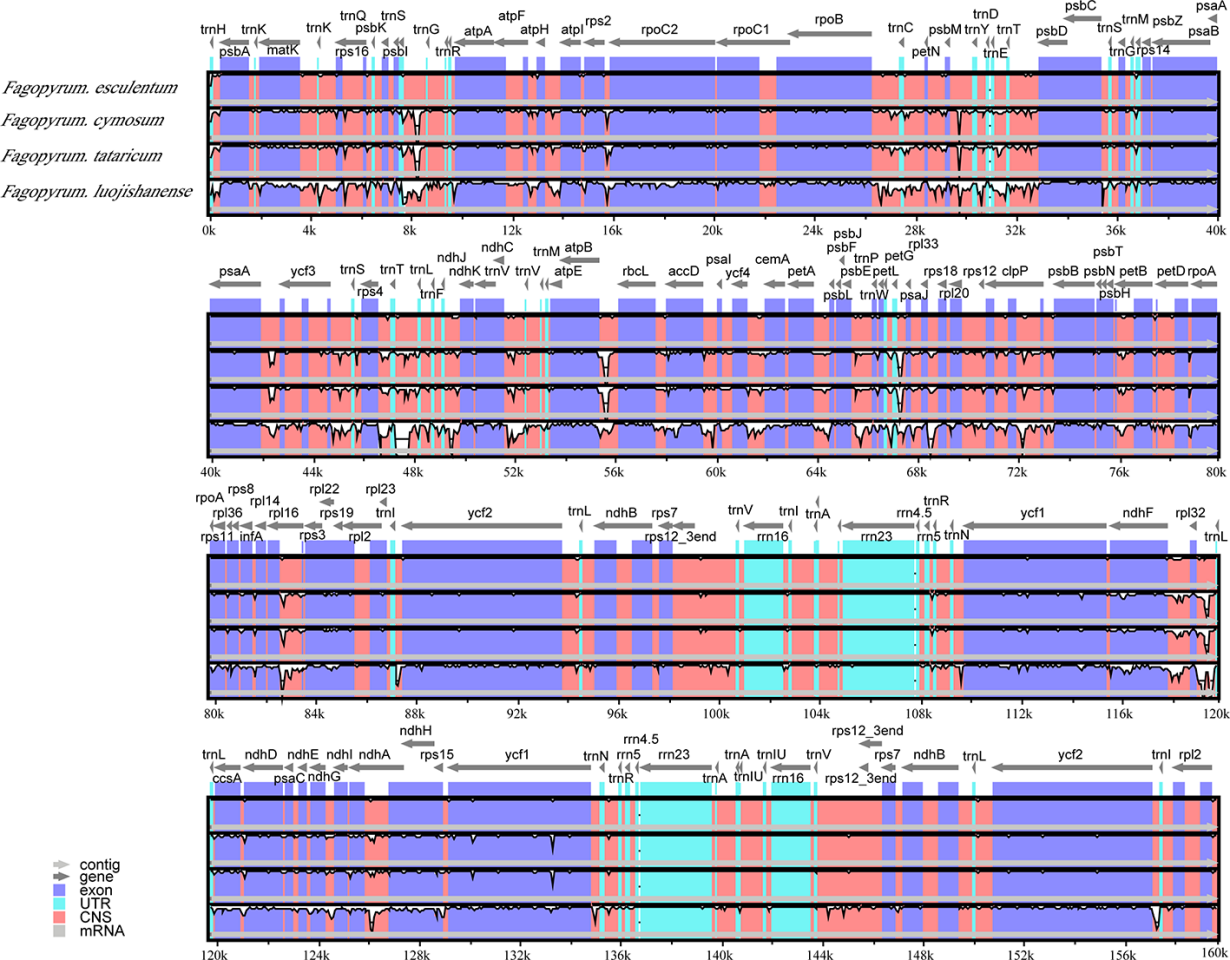
Sequence identity plots of five *Fagopyrum* chloroplast genomes by using mVISTA. The y-axis represents identity ranging from 50 to 100%.

The comparative account of cp genome and gene annotation studies confirmed that *F. esculentum* ssp*. ancestrale* is phylogenetically very close to *F. esculentum*. This was further confirmed by alignment analysis of five buckwheat, where divergence levels of cp genomes were found very low (**Figure 5**). Overall, the comparative account of cp genomes, revealed that *F. esculentum* ssp*. ancestrale* was highly similar to *F. esculentum*, and the chloroplast genome sequence of five buckwheat species was highly conserved.

### SSR Polymorphism in the cp Genomes of *F. esculentum* ssp. *ancestrale*, *F. cymosum* and *F. esculentum*

**Figure 6:**
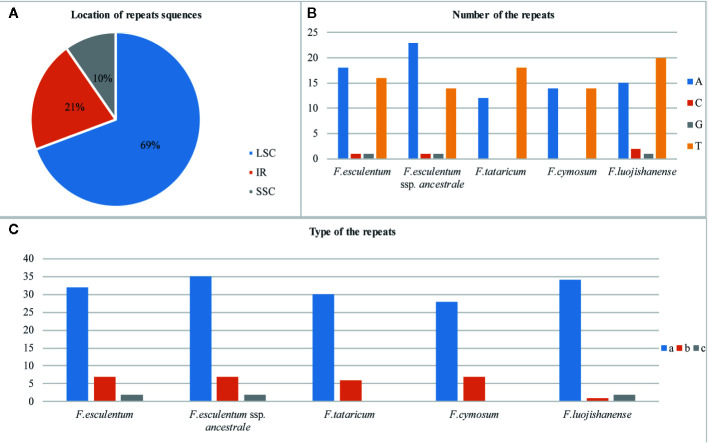
Overview of SSRs in the chloroplast genomes of five *Fagopyrum* species. **(A)** Distribution of SSRs in LSC, IR and SSC. **(B)** Number of nucleic acids in SSRs of five *Fagopyrum* species. **(C)** Type of the SSRs of five *Fagopyrum* species. a represents single base SSR, b represents composite SSR, c represents two base SSR.

The results revealed a total of 44, 35, and 41 SSRs in the *F. esculentum* ssp. *ancestrale*, *F. cymosum*, and *F. esculentum* cp genomes, respectively. An overview of cp genomes revealed that most of these SSRs were distributed in LSC followed by IR and SSC regions in all the three species (**Figure 6A**). While comparing the cp genomes of three *Fagopyrum* species, it was observed that *F. esculentum* ssp. *ancestrale* shares 27 and 31 identical SSR sequences with *F. cymosum* and *F. esculentum*, respectively. The most abundant SSRs motifs were mononucleotides, accounting for about 79.5, 80, and 78% of the SSRs motifs in *F. esculentum* ssp*. ancestrale*, *F. cymosum* and *F. esculentum*, respectively. Meanwhile, in all the three *Fagopyrum* species, the SSRs with A/T base repeat were significantly more than the G/C repeat. SSR types of *F. cymosum* and *F. tataricum* were similar, while the repeat types of *F. esculentum*, *F. luojishanense*, and *F. esculentum* ssp. *ancestrale* were closer, all containing the G/C type of SSR repeat sequences (**Figure 6B**). *F. esculentum* and *F. esculentum* ssp*. ancestrale* have similar type of SSR; Similar between *F. cymosum* and *F. tarticum*; *F. luojishanense* is a wild species and in the type of represents composite SSR number is the least compared to the others (**Figure 6C**). These results indicated that *F. esculentum* ssp. *ancestrale* was closer to *F. esculentum* than *F. cymosum*.

### Phylogenetic Analysis of *F. esculentum* ssp. *ancestrale*, *F. cymosum* and *F. esculentum* Based on cp Genome

**Figure 7:**
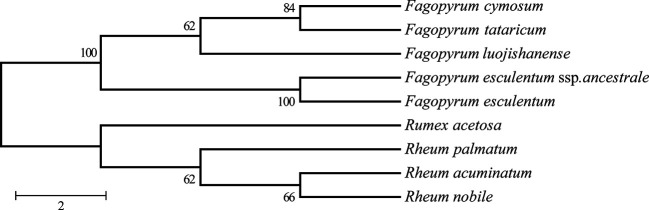
Phylogenetic relationships of the five *Fagopyrum* species inferred from MLanalysis constructed by chloroplast genome. This polygenetic tree was constricted by RA×ML and modified by MEGA and Adobe AI. The number on the branch displayed the bootstrap support values.

Comprehensive chloroplast genome data was utilized to construct a phylogenic tree to clarify the phylogenetic relationship between *F. esculentum* ssp. *ancestrale, F. cymosum* and *F. esculentum* (**Figure 7**). Along with five chloroplast genome *Fagopyrum* species, 4 out groups (**Table S3**) species were used to construct the phylogenic tree, which belongs to other genera in Polygonaceae family. based on these gene data a phylogenetic tree was constructed by using Three different methods i.e. MP/MB/ML. The findings signify that all the *Fagopyrum* species grouped together with very high internal resolution, and other four Polygonaceae species were gathering as the other cluster, Further the tree topology revealed that *F. esculentum* ssp. *ancestrale* grouped with *F. esculentum* with a high bootstrap score (**Figure 7**). To further investigate the evolutionary relationships, phylogenetic tree of all the 23 *Fagopyrum* species was constructed based on two chloroplast gene sequences (*mat*K and *trnhH-psbA*) and one nuclear gene sequence ITS). A perusal of tree topology based on ITS (**Figure S2**), *mat*K (**Figure S3**), and *trnhH-psbA* (**Figure S4**) revealed that *F*. *esculentum* ssp. *ancestrale* along with three cultivated species (*F. esculentum*, *F. tataricum* and *F. cymosum*) formed a single clade and delineated from rest of the buckwheat species. Further results showed that the *F. esculentum* ssp. *ancestrale* and *F. esculentum* grouped together in a sub-cluster revealing high homology and close hereditary relationship between the two species.

To further investigate the phylogenetic relationship among *F. cymosum*, *F. esculentum* ssp. *ancestrale*, and *F. esculentum*, PCR amplification using SSR markers BM469, BM463, and BM517 (Shi et al., 2015) were performed. SSR analysis revealed that *F. esculentum* ssp. *ancestrale* exhibits co-dominance with the bands amplified by *F. cymosum* and *F. esculentum* (**Figure S5**), supporting our hypothesis about the existence of a hybrid species (*F. esculentum* ssp. *ancestrale*) between the evolutionary route from *F. cymosum* to *F. esculentum*.

## Discussion

The three buckwheat species were characterized by leaf morphology, inflorescence type, flower type diversity, fruit color, and shape (**Table 2**). *F. esculentum* ssp. *ancestrale* was distinguishable to some extent from the two cultivated species on the basis of perianth color and fruit shape (**Table 2**). At the same time, *F. esculentum* ssp. *ancestrale* exhibits many similarities with *F. esculentum* and *F. cymosum*, especially with growth habit, petal, leaf, inflorescence type, and fruit color. The ambiguous patterns of morphological descriptors in all the three species support our hypothesis that *F. esculentum* ssp. *ancestrale* is a hybrid species between *F. esculentum* and *F. cymosum*. On the other hand, based on gross morphology and growth habit it is still hard to discriminate the three buckwheat species clearly. Furthermore, parallel and convergent evolution of morphological descriptors and strong dependence on environmental factors for full expression limit their utility for establishing phylogenetic links and distinctiveness among the closely related species and their hybrids (Mes et al., 1997). Therefore, determination of evolutionary relationships and distinctiveness among buckwheat species require more reliable tools like biochemical parameter, cp genome analysis, and molecular markers. There are two main advantages of their use in phylogenetic studies: (1) ease of observation and scoring; and (2) they are usually free from genotype-environment interaction. Additionally, speed of analysis, independence from the growth stage, location, season, and agronomy give them high value in phylogenetic analysis. These attributes which have been tested and confirmed, call for the consideration that biochemical parameters (flavonoids pattern), comparative cp genome analysis and molecular markers in combination with morphological descriptors will provide concrete genetic data for deducing phylogenetic link of three buckwheat species in our study.

### Flavonoids Concentration Established Phylogenetic Relationships Between Fagopyrum Species

Quantifying secondary metabolites have been used as a reliable method for species identification and determination of phylogenetic relationships between the related species of many plant genera (Bate-Smith and Richens, 1973; Stuessy and Crawford, 1983; Harris, 2009). For instance, the pattern of 21 flavonoids easily differentiated the 14 species of genus *crambe* of family *cruciferae* and also provided hints on the possible phylogenetic relationship between the species (Aguinagalde and Gomez-Campo, 1984). Recently, Zhuang and Tripp (2017) established a correlation between flavonoids pattern, phylogenetic relationships, and latitudinal spread of different species of the Neotropical genus *Ruellia*. These findings indicated that evolutionary history of different species within a genus is closely related to flavonoids pattern. In this study, we used total flavonoids, rutin and quercetin content for investigating the phylogenetic relationship of *F. esculentum* ssp. *ancestrale* with *F. esculentum* and *F. cymosum*. The total flavonoids content for *F. esculentum* ssp. *ancestrale* observed in the present study (5.0 mg/g) was considerably low than the total flavonoids content (13.0 mg/g) observed for *F. cymosum*, but very close to the total flavonoids content of (3.0 mg/100 g) reported for *F. esculentum*. The results have demonstrated the strong correspondence of evolutionary interpretations of these *Fagopryum* species flavonoids, which also supported our hypothesis of the phylogenetic status of *F. esculentum* ssp. *ancestrale*.

### Karyotype Analysis Revealed Close Genetic Proximity Between *F. esculentum* ssp. *ancestrale* and *F. esculentum*

Karyotype characterization is one of the most reliable methods for species differentiation and establishment of evolutionary relationship between different species of genus *Fagopyrum*. However, karyotype studies of *Fagopyrum* species through molecular cytogenetics are not thoroughly investigated and most of the findings were focused on traditional methods of determining the number and size of chromosomes (Neethirajan et al., 2011; Wang et al., 2017a). The diploid genomic constitution with 16 chromosomes of common buckwheat was first recognized by Taylor (1925). On the basis of morphology, most of the chromosomes of genus *Fagopyrum* are reported to be metacentric and no telocentric or acrocentric chromosomes have been perceived in any of the species (Zhou et al., 2012). Here, we expand this information with the results of FISH and rDNA analysis and demonstrate an upgraded methodology for karyotype analysis in buckwheat. In our study, the karyotype of *F. cymosum* (2n=2x=16) was reported to be with fifteen metacentric and one submetacentric chromosomes whereas, *F. esculentum* ssp. *ancestrale* (2n=2x=16) and *F. esculentum*(2n=2x=16) were represented by all the 16 metacentric chromosomes. Furthermore, the observed range of arm ratio (1.033–1.563) and proportion of longest to the shortest chromosome (1.967) for *F. esculentum* ssp. *ancestrale* were closely related to the arm ratio (1.119–1.406) and proportion of longest to the shortest chromosome (2.267) observed in *F. esculentum.* According to the karyotype classification of genomes (Stebbins, 1971), *F. cymosum* and *F. esculentum* ssp. *ancestrale* were appointed to class 1A, and *F. esculentum* was assigned to the class 1B karyoptype. Moreover, the 25s fluorescent mark revealed that there were six similar fluorescently labeled chromosomes in both *F. esculentum* and *F. esculentum* ssp. *ancestrale*, which were not found in the *F. cymosum*. Additionally, no microsatellites were observed in any of the chromosomes in *F. esculentum* ssp. *ancestrale* and *F. esculentum*. These similarities observed in chromosomal studies of *F. esculentum* ssp. *ancestrale* and *F. esculentum* provided strong evidence that they are genetically related to each other.

### Comparative Plastid Genomics Established Phylogenetic Link of *F. esculentum* ssp. *ancestrale* With *F. esculentum* and *F. cymosum*

The comparative analysis of extensively conserved chloroplast genomes has been utilized as a powerful molecular phylogenetic tool to establish evolutionary links between related species of many plant genera including *Fagopyrum* (Ohsako and Ohnishi, 2000; Zhou et al., 2014; Wang et al., 2017b). In general, chloroplast genomes are circular in shape ranging from 159 to 265 kb in length and comprised of LSC, SSC, and two copies of IR regions (Jansen and Ruhlman, 2012). In our study, chloroplast genomes of *F. esculentum* ssp. *ancestrale, F. cymosum and F. esculentum* were represented by a typical circular structure, which consisted of two copies of IR regions separated by the LSC and SSC region. The results highlighted that both IR regions have lower sequence divergence than LSC and SSC regions like in many other plant species, which is probably due to the gene conversion between IR sequences (Khakhlova and Bock, 2006).

According to their function, cp genes were divided into three categories, the first set of genes was related to transcription and translation, the second set was related to photosynthesis, the third set have a role in biosynthesis of amino acids, fatty acids as well as some genes with unknown functions. Analysis of our data could identify a total of 17 genes containing introns, 15 of which contain 1 intron and *ycf3*, and *clpP* contains 2 introns. *Rps12* is a specific *trans-shear* gene with its 5’ exon in the LSC region and the 3’ exon in the IR region. The comparative plastid genome analysis identified highly variable regions, including *trnS-trnG*, *rpoB-trnC*, *trnT-psbD*, *ycf3-trnS*, *trnT-trnL*, *rbcL-accD*, *ycf4-cemA*, *psbE-petL*, *ndhF-rpl32*, and *ndhA* introns. Sequence divergence in most of these regions located in LSC region have been reported as the molecular marker for phylogenetic relationship analysis in plant genera belonging to family *Lauraceae* (Song et al., 2015), *Leguminosae* (Drummond, 2008), *Solanaceae* (Levin et al., 2006) *Lamioideae* (Scheen and Albert, 2009), and Polygonaceae (Sanchez et al., 2009). Notably, the high nucleotide diversity reported in *ycf3-trnS* region and *ndhA* intron in this study is specific to *Fagopyrum*, which is in agreement with the results of Wang et al. (2017b). Unlike, the findings of Zhou et al. (2014), where highly variable sequence information pertaining to *mat*K *–* *tranK* region was used for phylogeny analysis of four wild species of genus *Fagopyrum* (*F. crispatifolium*, *F. pugense*, *F. qiangcai*, and *F. wenchuanense*) was found relatively conserved in our study. Overall, abundant molecular markers (SSRs and SNPs) can be generated from these highly variable regions for phylogenetic analysis and species identification in buckwheat.

The co-dominant mode of inheritance, hyper variability and high mutation rate of microsatellites (SSRs) make them potential markers for detecting polymorphism at the population level and phylogenetic relationships among species (Goldstein and Schlötterer, 1999). From our results, the most abundant SSRs in cp genomes were mononucleotide repeats followed by the di and trinucleotides. The numbers of mononucleotide repeats were almost similar in cp genomes of *F. esculentum* ssp.*ancestrale*, *F. esculentum* and *F. cymosum*. Furthermore, composite SSRs were reported in cp genome of *F. esculentum* ssp.*ancestrale* and *F. esculentum*, while they were absent in *F tataricum*. A comparative account of repeat sequences detected in LSC and SSC regions of *F. esculentum* ssp.*ancestrale* with *F. esculentum* and *F. cymosum* revealed that distribution pattern of majority of repeat sequences was same in all the three species. These observations further confirmed that *F. esculentum* ssp.*ancestrale* has a close phylogenetic link with *F. esculentum* and *F. cymosum*. The PCR amplification pattern of *F. cymosum*, *F. esculentum* ssp. *ancestrale* and *F. esculentum* using SSR markers BM 469, BM 460, and BM 517 (**Figure S5**), revealed that *F. esculentum* ssp. *ancestrale* exhibits co-dominance with the loci amplified by *F. cymosum* and *F. esculentum*, so we speculate that *Fagopyrum esculentum* ssp. *ancestrale* is a hybrid buckwheat species originated through spontaneous hybridization between *F. cymosum* to *F. esculentum* in their native habitat. Previously, utilizing AFLP, SSR, and allozyme variability, Ohnishi (2009) also established a close genetic and evolutionary link between the populations of common buckwheat and *F. esculentum* ssp. *ancestrale.*

## Conclusions

Studies based on floral and seed morphology, secondary metabolites, chloroplast genome analysis, and SSR banding pattern provided supporting evidence in favor of the hypothesis that *F. esculentum* ssp. *ancestrale* is originated by spontaneous hybridization between *F. cymosum* to *F. esculentum* under natural conditions.

## Data Availability Statement

The datasets presented in this study can be found in online repositories. The names of the repository/repositories and accession number(s) can be found in the article/**Supplementary Material**.

## Author Contributions

CC, YF, YT and RJ performed the experiments. KZ and MY analyzed sequencing data. MZ, DJ, and VM designed the research. DCJ, CC, and MZ wrote and revised the manuscript. All authors contributed to the article and approved the submitted version. There was no involvement of the funding providers in designing the study; collecting, analyzing, or interpreting the data; or deciding to submit the paper for publication. The authors have no conflict of interest affecting this paper.

## Funding

This work was supported by the National Key R&D Program of China (2017YFE0117600), the European Union Horizon 2020 project ECOBREED (771367), the National Natural Science Foundation of China (31871536), the Hunan Provincial Natural Science Foundation of China (2016JJ1010) and the Young Talent Supporting Plan of The Crop Science Society of China (2017QNRC182).

## Conflict of Interest

The authors declare that the research was conducted in the absence of any commercial or financial relationships that could be construed as a potential conflict of interest.
