# Supplementary material for: Fagopyrum esculentum ssp. ancestrale-A Hybrid Species Between Diploid F. cymosum and F. esculentum
Source: Front Plant Sci. 2020 Jul 16;11:1073. doi: 10.3389/fpls.2020.01073 (PMC7378737; doi:10.3389/fpls.2020.01073)
Supplement: Supplementary file 1 [file DataSheet_1.docx]

Table S1.*Fagopyrum* species used in this study

| wild species number | Latin | Collection place | Deposited location |
| --- | --- | --- | --- |
| 1 | *F. esculentum* ssp. *ancestrale* | Lijiang County, Yunnan Province | ICS, CAAS, BEIJING |
| 2 | *F. cymosum-Jianshui* | Jianshui County, Yunnan Province | ICS, CAAS, BEIJING |
| 3 | *F. cymosum-Hutiaoxia* | Shangri-La City, Yunnan Province | ICS, CAAS, BEIJING |
| 4 | *F. cymosum-Lijiang* | Lijiang City, Yunnan Province | ICS, CAAS, BEIJING |
| 5 | *F. cymosum-Luojishan* | Liangshan Prefecture, Sichuan Province | ICS, CAAS, BEIJING |
| 6 | *F. cymosum-Dali* | Dali City, Yunnan Province | ICS, CAAS, BEIJING |
| 7 | *F. cymosum-Sun* | Dali City, Yunnan Province | ICS, CAAS, BEIJING |
| 8 | *F. esculentum-Yuqiao* | Yulin City, Shanxi Province | ICS, CAAS, BEIJING |
| 9 | *F. esculentum-Zhongqiao Number One.01* | Beijing | ICS, CAAS, BEIJING |
| 10 | *F. tartaricum* | Liangshan Prefecture, Sichuan Province | ICS, CAAS, BEIJING |
| 11 | *F. Wild tataricum* | Aba Prefecture, Sichuan Province | ICS, CAAS, BEIJING |
| 12 | *F. wenchuanense* | Aba Prefecture, Sichuan Province | ICS, CAAS, BEIJING |
| 13 | *F. gracilipes var. odontopterum* | Liangshan Prefecture, Sichuan Province | ICS, CAAS, BEIJING |
| 14 | *F. luojishanense* | Liangshan Prefecture, Sichuan Province | ICS, CAAS, BEIJING |
| 15 | *F. rubifolium* | Aba Prefecture, Sichuan Province | ICS, CAAS, BEIJING |
| 16 | *F. capillatum* | Aba Prefecture, Sichuan Province | ICS, CAAS, BEIJING |
| 17 | *F. macrocarpum* | Aba Prefecture, Sichuan Province | ICS, CAAS, BEIJING |
| 18 | *F. lineare* | Dali City, Yunnan Province | ICS, CAAS, BEIJING |
| 19 | *F. urophyllum* | Lijiang City, Yunnan Province | ICS, CAAS, BEIJING |
| 20 | *F. leptopodum* | Liangshan Prefecture, Sichuan Province | ICS, CAAS, BEIJING |
| 21 | *F. jinshaense* | Lijiang City, Yunnan Province | ICS, CAAS, BEIJING |
| 22 | *F. gracilipes* | Liangshan Prefecture, Sichuan Province | ICS, CAAS, BEIJING |

Table S2. Primers nucleotide sequences and cycling conditions used for DNA analysis

| PCR region | Primer Sequences | Initial denaturation | No. of cycles | | Cycling parameters | | | Final extension | References |
| --- | --- | --- | --- | --- | --- | --- | --- | --- | --- |
|  |  |  |  | Denaturation | | Denaturation | Extension |  |  |
| ITS | F: TCC TCC GCT TAT TGA TAT GC  R: TCC GTA GGT GAA CCT GCG G | 94 °C  (5 min) | 35 | 94 °C  (30 s) | | 52.5°C  (45 s) | 72°C  (1 min) | 72 °C  ( 8 min) | Zheng et al. [24] |
| *mat*K | F: ATG GAG GAA TTC CAA GGA TAT TTA  R: TCA ATC ATT ATG ACT GGC CAA A | 94 °C  (5 min) | 35 | 94 °C  (30 s) | | 55 °C  (45 s) | 72°C  (90 s ) | 72 °C  ( 8 min) |  |
| *trnH-psbA* | F: CGC GCA TGG TGG ATT CAC AAT CC  R: GTT ATG CAT GAA CGT AAT GCT C | 94 °C  (5 min) | 35 | 94 °C  (1 min) | | 59 °C  (1 min) | 72°C  (1 min) | 72 °C  ( 8 min) |  |
| BM463 | F: AAA GAT GCC GAG ATA CAA CAG A  R: AGA AAT CGT GGA TGG GAG TTG G | 94 °C  (5 min) | 30 | 94 °C (30 s) | | 55 °C  (30 s) | 72°C  (1 min) | 72 °C  ( 7 min) | Shi et al.  [26] |
| BM469 | F: AGT CCC AGT CAT GGT CTC AAG C  R: TAC AAC AAG AAT GGA GGG AGG A |  |  |  |  |  |  |  |  |
| BM517 | F: TGG GAC CAA CAA GAG TGA CAA C  R: GAC CCG TGC TTT ACT CTT TAC C. |  |  |  |  |  |  |  |  |

Table S3. Genomic information used in phylogenetic analysis based on chloroplast genome

| Section name | Genus name | Species name | GeneBanK Number |
| --- | --- | --- | --- |
| Polygonaceae | Fagopyrum | *Fagopyrum esculentum* | EU254477 |
| Polygonaceae | Fagopyrum | *Fagopyrum tataricum* | KM201427 |
| Polygonaceae | Fagopyrum | *F. cymosum* | KY275181 |
| Polygonaceae | Fagopyrum | *F. luojishannse* | KY275182 |
| Polygonaceae | Rheum | *Rheum palmatum* | KR816224 |
| Polygonaceae | Rumex | *Rumex acetosa* | KC817303 |
| Polygonaceae | Rheum | *Rheum nobile* | NC_046506.1 |
| Polygonaceae | Rheum | *Rheum acuminatum* | MN514858.1 |

Table S4. Genomic information used in hierarchical cluster analysis based on ITS, *mat*K, *trnhH-psbA*.

| wild species number | Latin | ITS GeneBanK Number | *mat*K GeneBanK Number | *trnhH-psbA* GeneBanK Number |
| --- | --- | --- | --- | --- |
| 1 | *F. esculentum* ssp. *ancestrale* | MT644598 | MT668917 | MT668925 |
| 2 | *F. cymosum-Jianshui* | HM357907.1 | AB093078.1 | NC_037705.1 |
| 3 | *F. cymosum-Hutiaoxia* | JF708196.1 | AB093074.1 | MF491390.1 |
| 4 | *F. cymosum-Lijiang* | AB000327.1 | JF829983.1 | KY206921.1 |
| 5 | *F. cymosum-Luojishan* | JN083791.1 | JN012073.1 | KY275181.1 |
| 6 | *F. cymosum-Dali* | FJ503008.1 | AB093081.1 | MH196562.1 |
| 7 | *F. cymosum-Sun* | AB000324.1 | AB093075.1 | EU554044.1 |
| 8 | *F. esculentum-Yuqiao* | KY945281.1 | JN012075.1 | EF653736.1 |
| 9 | *F. esculentum-Zhongqiao Number One.01* | KY945315.1 | EU840460.1 | KY206922.1 |
| 10 | *F. tartaricum* | KY945293.1 | JN012074.1 | KX085498.1 |
| 11 | *F. Wild tataricum* | KY945296.1 | JF829984.1 | KY206928.1 |
| 12 | *F. wenchuanense* | JF829996.1 | JF829982.1 | JQ807576.1 |
| 13 | *F. gracilipes var. odontopterum* | MT672487 | JF829974.1 | MT668927 |
| 14 | *F. luojishanense* | MT644599 | MT668923 | MT668928 |
| 15 | *F. rubifolium* | MT644600 | MT668924 | MT668929 |
| 16 | *F. capillatum* | AB000323.1 | MT668920 | MT668930 |
| 17 | *F. macrocarpum* | MT644601 | MT668921 | MT668931 |
| 18 | *F. lineare* | AB000335.1 | MT668922 | KY206926.1 |
| 19 | *F. urophyllum* | AB000342.1 | MT668919 | KY206929.1 |
| 20 | *F. leptopodum* | AB000334.1 | JF829978.1 | KY206925.1 |
| 21 | *F. jinshaense* | MT644602 | MT668918 | MT668926 |
| 22 | *F. gracilipes* | AB000332.1 | JN012076.1 | KY206924.1 |
| 23 | *Polygonum bistorta* | FJ503009.1 | AF204859.1 | EU554046.1 |


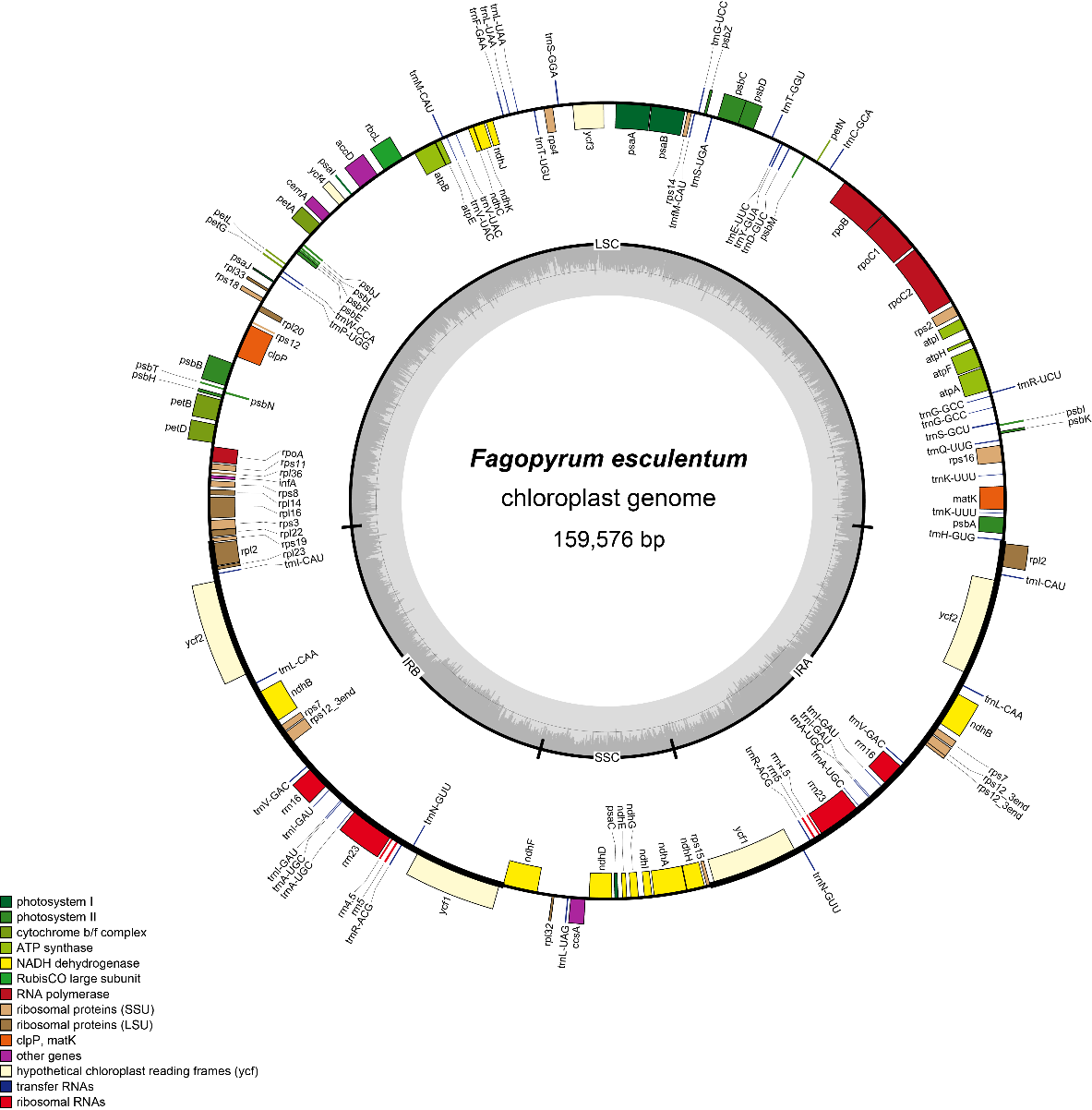


Fig S1. Gene map of *F. esculentum*. The genes shown outside of the circle are transcribed clockwise, while those inside are counte clockwise.


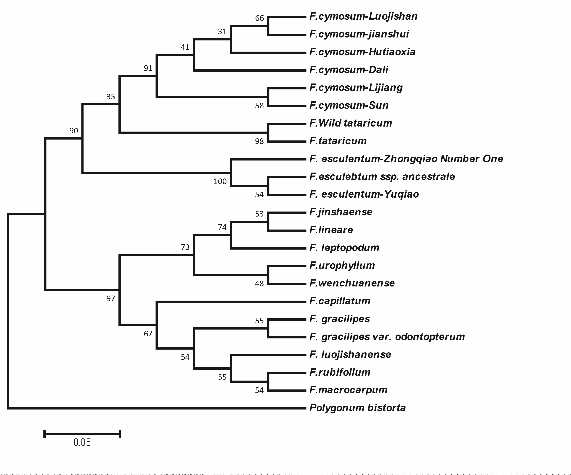


Fig S2. Dendrogram of 23 Fagopyrum species based on ITS using hierarchical cluster analysis


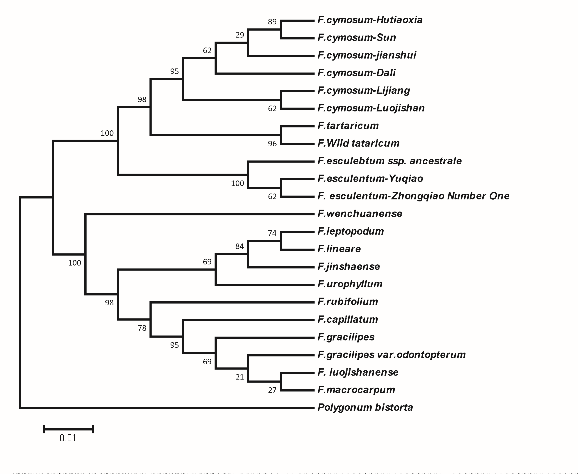


Fig S3. Dendrogram of 23 Fagopyrum species based on *mat*K using hierarchical cluster analysis


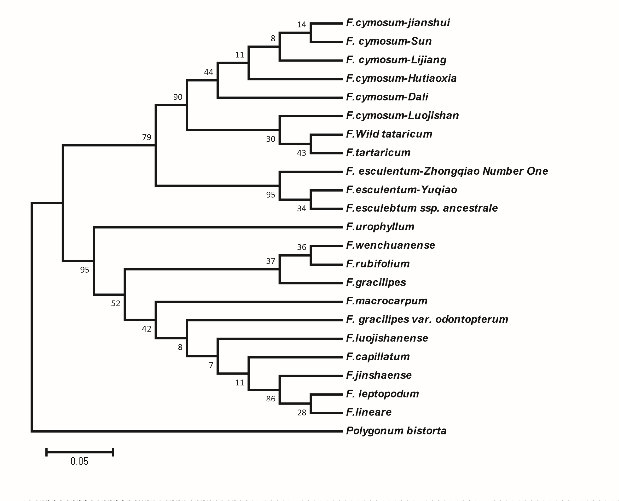


FigS4. Dendrogram of 23 Fagopyrum species based on *trnhH-psbA* using hierarchical cluster analysis


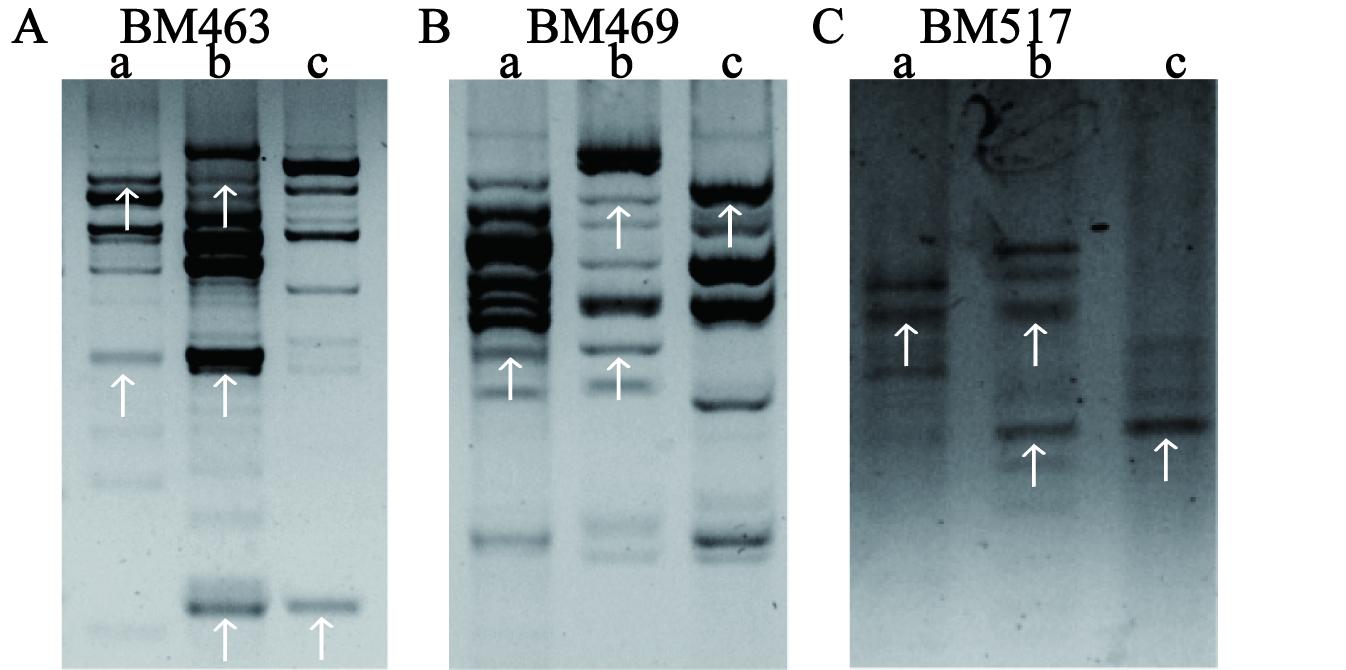


Fig S5. Polymorphism between three buckwheat species using SSR molecular markers, a: *F. cymosum,* b: *F. esculentum* ssp*. ancestrale,* c: *F. esculentum*
